# Investigating the Relationships of P3b with Negative Symptoms and Neurocognition in Subjects with Chronic Schizophrenia

**DOI:** 10.3390/brainsci11121632

**Published:** 2021-12-10

**Authors:** Giulia M. Giordano, Andrea Perrottelli, Armida Mucci, Giorgio Di Lorenzo, Mario Altamura, Antonello Bellomo, Roberto Brugnoli, Giulio Corrivetti, Paolo Girardi, Palmiero Monteleone, Cinzia Niolu, Silvana Galderisi, Mario Maj, The Italian Network for Research on Psychoses

**Affiliations:** 1 Department of Psychiatry, University of Campania “Luigi Vanvitelli”, 80138 Naples, Italy; giuliamaria.giordano@unicampania.it (G.M.G.); andrea.perrottelli@unicampania.it (A.P.); silvana.galderisi@unicampania.it (S.G.); mario.maj@unicampania.it (M.M.); 2 Department of Systems Medicine, University of Rome “Tor Vergata”, 00133 Rome, Italy; di.lorenzo@med.uniroma2.it (G.D.L.); niolu@med.uniroma2.it (C.N.); 3 Psychiatry Unit, Department of Clinical and Experimental Medicine, University of Foggia, 71122 Foggia, Italy; mario.altamura@unifg.it (M.A.); antonello.bellomo@unifg.it (A.B.); 4 Department of Neurosciences, Mental Health and Sensory Organs, S. Andrea Hospital, University of Rome “La Sapienza”, 00189 Rome, Italy; roberto.brugnoli@uniroma1.it (R.B.); paolo.girardi@uniroma1.it (P.G.); 5 Department of Mental Health, University of Salerno, 84133 Salerno, Italy; corrivetti@gmail.com; 6 Section of Neurosciences, Department of Medicine, Surgery and Dentistry, ‘Scuola Medica Salernitana’, University of Salerno, 84081 Salerno, Italy; pmonteleone@unisa.it

**Keywords:** schizophrenia, neurocognition, negative symptoms, EEG, P3b

## Abstract

Neurocognitive deficits and negative symptoms (NS) have a pivotal role in subjects with schizophrenia (SCZ) due to their impact on patients’ functioning in everyday life and their influence on goal-directed behavior and decision-making. P3b is considered an optimal electrophysiological candidate biomarker of neurocognitive impairment for its association with the allocation of attentional resources to task-relevant stimuli, an important factor for efficient decision-making, as well as for motivation-related processes. Furthermore, associations between P3b deficits and NS have been reported. The current research aims to fill the lack of studies investigating, in the same subjects, the associations of P3b with multiple cognitive domains and the expressive and motivation-related domains of NS, evaluated with state-of-the-art instruments. One hundred and fourteen SCZ and 63 healthy controls (HCs) were included in the study. P3b amplitude was significantly reduced and P3b latency prolonged in SCZ as compared to HCs. In SCZ, a positive correlation was found between P3b latency and age and between P3b amplitude and the Attention-vigilance domain, while no significant correlations were found between P3b and the two NS domains. Our results indicate that the effortful allocation of attention to task-relevant stimuli, an important component of decision-making, is compromised in SCZ, independently of motivation deficits or other NS.

## 1. Introduction

Neurocognitive deficits and negative symptoms have a pivotal role in schizophrenia due to their severe impact on patients’ functioning in daily life and the absence of effective pharmacological treatments targeting them [1,2,3,4,5,6,7,8,9,10,11,12,13,14].

A mild to severe impairment in neurocognitive skills is present in the majority of subjects with schizophrenia, independently of the severity of symptoms. Furthermore, this impairment can be detected throughout different phases of schizophrenia, such as the premorbid, prodromal and remission stages of the illness or even in non-affected relatives of subjects with schizophrenia [15,16,17,18,19,20,21,22,23,24,25,26,27]. This deficit affects several neurocognitive domains, such as attention, speed of processing, working memory, visuospatial learning and memory, verbal learning and memory, reasoning, problem solving and executive functions (e.g., the ability to plan, execute and monitor goal-directed behavior in a flexible and adaptive way) [8,28,29]. From a neurobiological perspective, impairments in these functions might be explained as the cumulative effect of abnormalities in processes such as early neurodevelopment, neuronal maturation and neuroplasticity, thus leading to faulty cortico–cerebellar–thalamic–cortical circuits [30,31,32,33,34,35,36].

As regard to negative symptoms, according to the current conceptualization, they include blunted affect (reduced intensity and range of emotional expression), alogia (reduced spontaneous speech and loss of conversational fluency), avolition (reduced interest and motivation for goal-directed activities), asociality (diminished social drive or interest and desire for affiliation) and anhedonia (reduced ability to experience or anticipate pleasure) [37,38,39,40]. These symptoms cluster into two domains, the Expressive one, which includes blunted affect and alogia, and the Experiential domain, which includes avolition, anhedonia and asociality [38,40,41,42,43,44,45]. The most updated hypothesis underlying negative symptoms indicates a relationship between the Experiential domain and deficits in different aspects of motivation, which are fundamental for the achievement of goal-directed decision-making [40,42,46,47,48,49,50,51,52,53,54,55,56,57,58,59,60,61,62,63,64,65,66,67,68]. These compromized aspects include abnormalities in reward prediction (the ability to predict a pleasant experience), value encoding (the ability to determine current value of a stimulus in the context of a motivational state), action outcome contingency learning (the ability to know the causal consequences of an action) and in the integration of goal-directed behavior and experienced value [40,61,62,63,69,70,71]. Motivational deficits might also be due to abnormalities in orientation towards salient stimuli (aversive or rewarding stimuli), cognitive activation and general motivation [40,69,70,72]. Another hypothesis states that Experiential domain symptoms, in particular avolition, are actually caused by deficits in the executive control of behavior. Indeed, it has been demonstrated that deficits in plan elaboration might impair goal-directed behaviour, with subsequent reduced motivation and increased negative symptoms of the experiential type, i.e., avolition, anhedonia and asociality [40,65,73,74]. However, findings across studies are inconsistent and need further investigation [40,65,73,74,75,76].

Intuitively, deficits in different aspects of motivation and neurocognitive skills, for instance in executive functions and in the allocation of attentional resources to relevant stimuli, might hinder decision making and goal-directed behavior [77,78]. However, it is not clear whether these processes share common pathophysiological mechanisms or whether they affect goal-directed decisions through different neuronal pathways. Furthermore, studies using reward-related tasks do not discriminate between different aspects of decision-making deficits: those related to the allocation of resources to task-relevant stimuli, which do not involve motivational deficits, and those deriving from impaired reward processing, including valuation, learning of reward–outcome associations, which also can impair decision making.

In the context of schizophrenia research, electrophysiological (EEG) recordings have repeatedly been employed to investigate the neurobiological correlates of cognitive impairment and negative symptoms [79,80]. Specifically, the analysis of event-related potentials (ERPs) represents an objective tool to decipher mental processes, due to its high temporal resolution in capturing responses to internal and external events [81,82].

One of the ERPs most extensively studied is P300, a positive-going deflection peaking between 270 and 600 msec, which can be identified following the presentation of infrequent auditory, visual or somatosensory stimuli [83,84]. In addition, P300 can be recorded using experimental paradigms focusing on decision-making and risk evaluation [85], motivation and anticipation of reward [86,87,88,89], perception of emotions [90,91] and allocation of attentional resources [92]. Different subcomponents of P300 have been categorized, which present distinct neural generator sources, topographic maps and peak latencies depending on the paradigm used [92,93,94]. Two main subtypes have been characterised: P3a and P3b [92]. The former can be recognised for its scalp distribution with a fronto-central maximum and its elicitation in response to deviant and unexpected events, regardless of their task-relevance. It is therefore often been categorised as a marker of saliency and unconscious attentional capture [92]. Conversely, P3b has a more parietal distribution and is linked to sensory processing of a rare stimulus that is task-relevant, i.e., requiring subject’s response.

In psychiatric research, P300 started to gain attention due to the consistent alterations recorded in subjects with schizophrenia. In fact, numerous studies have reported a reduction in P300 amplitude and delayed latency of its peak both in subjects with chronic schizophrenia [95,96,97] and in subjects at prodromal and early stages of the illness [98].

Furthermore, P3b has been regarded as a candidate biomarker of neurocognitive impairment, due to its linkage in physiological conditions to vigilance, allocation of attentional resources, direct updating of the stimulus representation and executive functions [83]. In addition, it has also been observed that increasing age is associated with alterations of P3b probably due to cognitive decline linked to aging [99,100,101].

Previous studies in schizophrenia have observed a direct association between deficits in the elicitation of P3b and the severity of impairments in attention [102,103,104,105], executive functions [102,106,107], memory [105,107,108,109,110,111,112] and verbal learning [112,113,114]. However, other studies did not report any correlations between P3b amplitude and neurocognitive impairment in subjects with schizophrenia [113,115,116,117]. These inconsistent findings might be due to differences across studies in sample sizes, characteristics of included patients, as well as in instruments used to assess neurocognitive impairment.

In addition, some studies found that reductions in P3b amplitude were correlated to negative symptoms severity in subjects with chronic schizophrenia [118,119,120] and in subjects at-risk for psychosis [121,122,123]. One study reported a relationship between the improvement in negative symptom severity scores and the increase of P300 amplitude following antipsychotic medications [124]. Conversely, some studies did not report any associations between P3b and negative symptoms [88,125,126,127]. In the study of Vignapiano et al. [88], the authors found that P300 amplitude for large reward and large loss was inversely related to trait anhedonia.

It is important to point out that the majority of the above-mentioned studies [118,119,123,124,125,126] evaluated negative symptoms using first generation rating scales, such as the Positive and Negative Syndrome Scale [128], the Scale for the Assessment of Negative Symptoms (SANS) [129] and the Brief Psychiatric Rating Scale (BPRS) [130]. However, these scales present some limitations, as they include aspects that actually are not conceptualized as negative symptoms, but are mostly related to cognitive functions and disorganization [38].

The association between P300 abnormalities with clinical and cognitive aspects in subjects with schizophrenia is still a matter of debate due to the inconsistent findings reported in the literature.

Our study aims to investigate in a large sample of subjects with schizophrenia the associations of P3b with multiple cognitive domains and the expressive and motivation-related domains of NS, evaluated with state-of-the-art instruments.

To pursuit this aim, the study investigated: (1) the differences in P3b parameters between subjects with schizophrenia and healthy controls; (2) the associations between P3b parameters with neurocognitive functions both in patients and healthy controls; (3) the relationship between P3b parameters and negative symptoms in subjects with schizophrenia.

## 2. Materials and Methods

### 2.1. Study Participants

The study has been conducted as part of the add-on EEG study of the Italian Network for Research on Psychoses [2]. One hundred and forty-eight subjects with schizophrenia (SCZ) and 70 healthy controls (HCs) were recruited for the study, at five research sites in Naples, Foggia, Rome Tor Vergata, Rome “Sapienza” and Salerno. The SCZ sample included individuals seen at the outpatient units of the five mentioned Italian university psychiatric clinics. All patients had a diagnosis of schizophrenia according to DSM-IV, confirmed with the Structured Clinical Interview for DSM IV-Patient version (SCID-I-P), and an age between 18 and 65 years.

The HCs sample was recruited from the community at the same sites mentioned above. Inclusion criteria for HCs were the absence of a current or lifetime Axis I or II psychiatric diagnosis. Exclusion criteria for both groups were: (a) a history of head trauma with loss of consciousness; (b) a history of moderate to severe mental retardation or of neurological diseases; (c) a history of alcohol and/or substance abuse in the last six months; (d) current pregnancy or lactation; and (e) inability to provide an informed consent. SCZ with treatment modifications and/or hospitalization due to symptom exacerbation in the last three months were excluded.

The Ethics Committee of the involved institutions approved the electrophysiological add-on study. The study has been performed in accordance with the ethical standards laid down in the 1964 Declaration of Helsinki. All participants signed a written informed consent to participate after receiving a detailed explanation of the study procedures and goals.

### 2.2. Clinical and Neurocognitive Assessments

All subjects recruited were evaluated for sociodemographic variables such as age, education and gender, through a clinical form filled using every available source of information.

For SCZ, a semi-structured interview, the Brief Negative Symptom Scale (BNSS) was used to assess negative symptoms [131,132]. The scale includes 13 items, organized into six subscales (Blunted Affect, Alogia, Avolition, Anhedonia, Asociality and a control subscale named Distress). All the items use a seven-point (0–6) scale, thus ranging from absent (0) to moderate (3) to extremely severe (6) symptoms (except Distress for which the severity rating is reversed: 0 normal distress and 6 absent).

With regard to the two domains, the Experiential domain was computed by summing the scores on the subscales Anhedonia (consummatory and anticipatory anhedonia), Avolition and Asociality; the Expressive deficit was computed by summing the scores on the subscales Blunted Affect and Alogia [131].

The Positive and Negative Syndrome Scale (PANSS) was used to rate the severity of positive symptoms and disorganization [128]. All items are rated on a seven-point scale from 1 to 7, ranging from absent (1) to moderate (4) to extremely severe (7). We also assessed depressive symptoms using the Calgary Depression Scale for Schizophrenia (CDSS) [133] and extrapyramidal symptoms using the St. Hans Rating Scale (SHRS) [134].

Neurocognitive domains were assessed in SCZ and HCs using the Measurement and Treatment Research to Improve Cognition in Schizophrenia (MATRICS) Consensus Cognitive Battery (MCCB) [135]. This battery includes tests for the assessment of distinct neurocognitive domains: speed of processing, attention/vigilance, working memory, verbal learning and memory, visuospatial learning and memory, reasoning and problem solving.

Raw scores on the MCCB were then standardized to T-scores, corrected for age and gender, based on the Italian normative sample of community participants.

### 2.3. EEG Recording Procedure

EEGs were recorded using two highly comparable EEG recording systems: EASYS2 (Brainscope, Prague) and Galileo MIZAR-sirius (EBNeuro, Florence). Before starting the study, a harmonization of the amplifier settings and recording procedure was carried out to ensure the same settings in all the centers. All EEGs were recorded using a cap electrode system with 29 unipolar leads (Fpz, Fz, Cz, Pz, Oz, F3, F4, C3, C4, FC5, FC6, P3, P4, O1, O2, Fp1, Fp2, F7, F8, T3, T4, T5, T6, AF3, AF4, PO7, PO8, Right Mastoid and Left Mastoid), which were placed following the 10–20 system. All the leads were referenced to the linked earlobes (a resistor of 10 kΩ was interposed between the earlobe leads). A ground electrode was placed on the forehead.

For artifact monitoring, a horizontal electro-oculogram (hEOG) was recorded from the epicanthus of each eye, and a vertical EOG (vEOG) from the leads beneath and above the right eye. All impedances of the leads were kept below 5 kΩ. The EEG data were filtered with a band-pass of 0.15–70 Hz and recorded with a sampling rate of 512 Hz.

A calibration was performed for all channels, using a 50 μV sine wave, before each recording session. Subjects were seated in a reclining chair, in a sound attenuated room, minimizing eye movement or muscle tension. Subjects performed an auditory “odd-ball” task during which 320 standard stimuli (1500-Hz, 80 dB) and 80 target stimuli, deviant for their frequency (1000-Hz, 80 dB), were played. Patient were asked to press the button as fast as possible upon the appearance of every target stimulus. Participants who scored less than 60% on the behavioral target detection task were excluded from the analysis.

Participants were instructed not to drink coffee or tea and to abstain from smoking cigarettes in the 2 h before the beginning of the recording session and did not take psychotropic medications in the morning. Information on the quality of sleep during the night prior to the recording was collected and the EEG session was postponed if the subject reported a non-restoring sleep.

### 2.4. EEG Data Preprocessing

One expert from the coordinating center (Naples) using Brain Vision Analyzer software (Brain Products, Munich, Germany) performed all the pre-processing analyses on data collected by the different recording sites. In order to characterize P3b deflections, we considered only the 80 trials when target stimuli were played. Data were parsed into epochs of 1000 ms duration, which were time-locked to the onset of the cue and spanned from a 100 ms pre-stimulus period up to 900 msec post-stimulus. The recorded EEG was digitally filtered offline using a band-pass filter of 0.01–30 Hz. P3b waves were extracted in each subject by the averaging method on all the “target” trials, in order to improve the signal/noise ratio, ruling out baseline activity not related to the stimulus. Trials with drifts larger than ±100 μV in any scalp electrode were rejected. If following artifacts and noisy trials removal, less than 40 usable target trials (50% of target trials) remained, the subject was excluded from the analysis. Data were baseline-corrected using the 100 ms time window preceding stimuli. P3b peaks were automatically marked using the “peak finder” function of Brain Analyzer, as the most positive point ranging from 240 to 480 ms. Due to its prevalent parietal localization, we analyzed only amplitude and latency of P3b from the Pz electrode [105,136].

### 2.5. Statistical Analysis

All statistical analyses were computed using SPSS Version 22.0 (Armonk, NY, USA: IBM Corporation, 2014). Normality tests were performed on demographic, clinical and electrophysiological variables to test distribution of data in order to set up parametric or non-parametric tests.

Two sample *t*-tests, Mann–Whitney U Tests and χ^2^ tests were used to compare SCZ and HCs on demographic characteristics, MCCB scores and amplitude and latency of P3b, based on normality test results. Spearman’s rank correlations were performed to test the relationships between P3b parameters with age, neurocognitive domains (separately for the two sample groups) and the two negative symptom domains in SCZ. For all the correlations considered, Bonferroni–Holm correction was applied in order to control for type-I error inflation. Furthermore, we performed partial correlations to exclude the influence of other variables (for negative symptoms we decided to control the effect of age, positive symptoms, disorganization, depression and parkinsonism; for neurocognitive impairment the effect of age and education).

## 3. Results

### 3.1. Participants Included

One hundred and forty-eight SCZ and 70 HCs were originally enrolled as part of the add-on EEG study. However, 23 SCZ and 4 HCs did not complete the paradigm for P3b recording. Furthermore, 11 SCZ and 3 HCs were excluded either for the high presence of artefacts in the ERP recordings or for low behavioural performance on the active target recognition task. Thus, the final study sample consisted of 114 SCZ and 63 HCs.

### 3.2. Demographic Characteristics, Neurocognitive Functions and Illness Related Variables

Data on relevant demographic characteristics, neurocognitive functions and illness related variables are provided in Table 1. Gender distribution was significantly different between the two groups (χ^2^ = 7.214; *p* < 0.01) since in the SCZ group the number of male subjects was higher, as compared to HCs. There was no significant difference in the mean age between the two sample groups (U = 2982.00; *p* > 0.05). Furthermore, not unexpectedly, SCZ had significantly lower education as compared to HCs (*p* < 0.01). The mean of MCCB domain scores are shown in Table 1. The two groups significantly differed for the scores recorded on all six neurocognitive domains (*p* < 0.0001), with SCZ showing a noticeable worse performance on all the evaluated domains.

### 3.3. Group Comparison on P3b Amplitude and Latency

**Figure 1:**
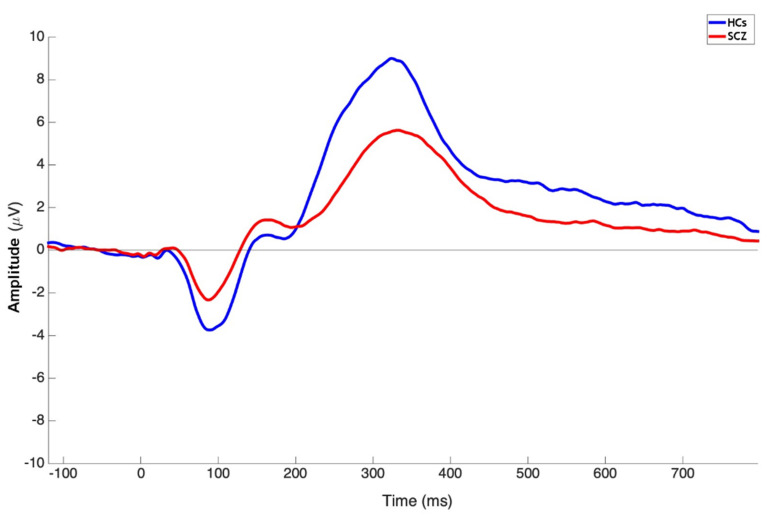
Grand average ERP waveforms of subjects with schizophrenia (red line) and healthy controls (blue line) from the Pz electrode.

SCZ showed reduced P3b amplitude and delayed latency as compared to HCs (Figure 1).

Mann–Whitney U tests showed that there was a significant difference in P3b amplitude (U = 2023.00; *p* < 0.0001) and latency (U = 2683.50; *p* = 0.0054) between SCZ and HCs as recorded at Pz (Table 2).

### 3.4. Correlation Analyses

**Figure 2:**
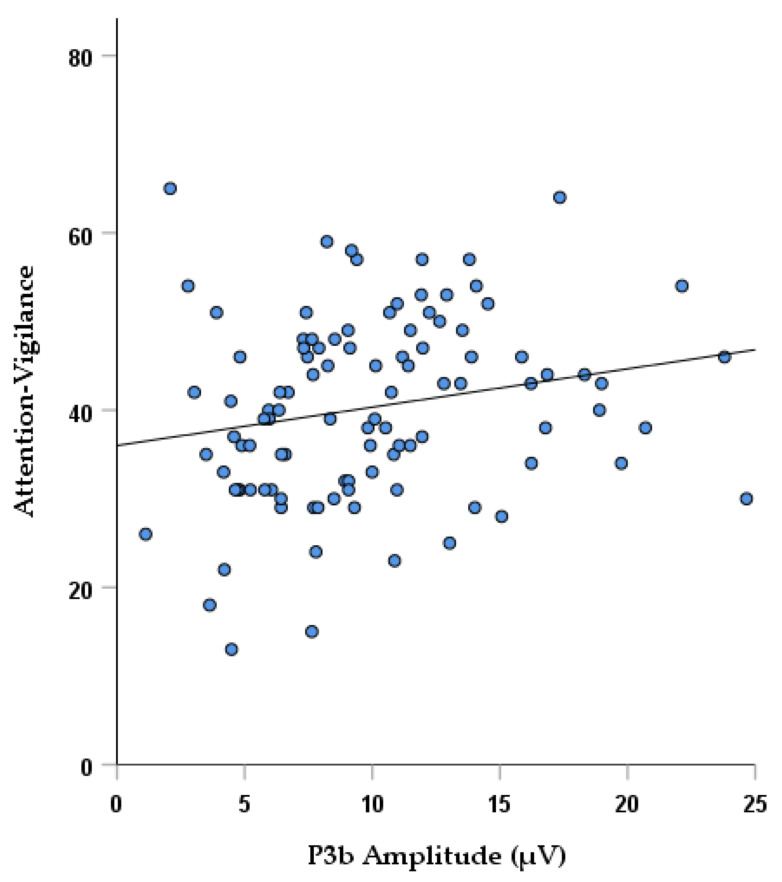
Correlation between P3b amplitude and attention-vigilance domain scores in SCZ.

Considering neurocognitive functioning, a significant correlation was found in SCZ between P3b amplitude and the attention-vigilance domain scores (*r*_s_ = 0.259; *p* = 0.0076), which remained significant even when controlling for age and years of education (Table 3; Figure 2). Furthermore, a negative correlation was observed at trend level between P3b latency and verbal (*r*_s_ = −0.223; *p* = 0.019) and visuo-spatial learning and memory (*r*_s_ = −0.195; *p* = 0.043), which did not survive correction for multiple tests (Table 3). In SCZ, a significant positive correlation was found between P3b latency and age (*r*_s_ = 0.320; *p* = 0.00052) (Table 3). No significant correlations (*p* > 0.05) were found between P3b amplitude or latency and the two negative symptom domains.

In HCs, neither of the two P3b parameters was associated with the six cognitive domains considered. However, age was negatively correlated with P3b amplitude (*r*_s_ = −0.470; *p* = 0.00010), and positively with P3b latency (*r*_s_ = −0.309; *p* = 0.014) (Table 4).

### 3.5. Control Analyses

Additional control tests were performed. Firstly, regression models were designed to check if P3b amplitude/latency could predict neurocognition scores controlling for group, education and age (Supplementary Material). Multiple regression models showed that T-Scores for two neurocognitive domains could be predicted from P3b values. Specifically, Attention-vigilance domain scores (*R*^2^ = 0.256, *p* < 0.001) could be predicted from group, education and P3b amplitude, while Visual (*R*^2^ = 0.167, *p* < 0.001) learning scores were predicted by group and P3b latency. Furthermore, in both regression models no significant interaction effects (*p* > 0.05) were detected between the electrophysiological features and the other significant predictors. Secondly, in order to remove the effects of normal aging, while allowing disorder-relevant aging effects to remain, additional correlation tests employing age-corrected P3b amplitude and latency z-scores were performed (Supplementary Tables S1 and S2). Results showed that the significant correlation between P3b amplitude and Attention-vigilance was still detectable in SCZ as well as a weaker, but still significant, negative correlation was recorded between P3b latency and Verbal Learning scores (Supplementary Table S1). Furthermore, also in these control correlation analyses, no significant correlations emerged with negative symptom domains. Finally, we also tested if P3b values in SCZ could be possibly related to depression (CDSS Total scores) (Supplementary Material), but no significant correlation emerged (*p* > 0.05).

## 4. Discussion

The current study aimed to examine auditory-elicited P3b in schizophrenia, disentangling its association with neurocognitive impairment and negative symptoms. Three main objectives were addressed through data analysis: (1) To identify differences in P3b amplitude and peak latency between subjects with schizophrenia and healthy controls; (2) to investigate the presence of associations between P3b parameters with neurocognitive domains in subjects with schizophrenia and healthy controls; and (3) to detect correlations between P3b characteristics and severity of the two domains of negative symptoms in subjects with schizophrenia.

The outcomes of the analysis revealed a reduction in P3b amplitude and a delayed peak in response to target stimuli in subjects with schizophrenia as compared to healthy controls. From the correlation analysis, a statistically significant association between diminished P3b amplitude and lower scores in the attention-vigilance domain was found in subjects with schizophrenia. Additionally, in the same subjects a trend-level association was reported between P3b latency and verbal and visual learning. No significant relationship emerged in healthy controls between P3b parameters and cognitive functions, probably due to the existence of a marked cognitive impairment present only in subjects with schizophrenia. Nonetheless, the control analysis using multiple regression models, which combined the two sample groups, revealed that for the Attention-vigilance and visual learning T-scores, lower amplitude and delayed latency in P3b were related to worse cognitive performance, in the whole study sample. This suggest that the slopes of the equations predicting Attention-vigilance and Visual learning scores from P3b values were not significantly different between the two groups. However, the lack of significant associations in the main correlation analysis between P3b and neurocognitive domains in healthy subjects might be due to two factors. One possible explanation might be that the lower number of subjects included in the HCs group has hindered the emergence of significant correlations. Secondly, the majority of the T-scores values for this group were distributed only in the medium-high range of the values spectrum, which might lead to the absence of a robust correlation between P3b and cognitive performance. This reinforces the hypothesis that the association between P3b features and neurocognition might be present and detectable mainly in pathological conditions.

Furthermore, as regard to the possible association between P3b and age, we found in both sample groups that P3b peak latency was proportionally delayed as age increased. A statistically significant relationship between P3b amplitude reductions and increasing age was found in healthy subjects, while an association at trend-level was found in subjects with schizophrenia. Furthermore, the control analyses have also reinforced the finding that the strong association of P3b attenuation with aging was not driving the correlation with attentive impairment present in subjects with schizophrenia. Finally, no associations between negative symptom domains and P3b measures emerged in subjects with schizophrenia in the main and control analyses.

Our findings of altered P3b values in patients, displayed as reduced amplitude and delayed latency, are in agreement with the vast literature produced on this EEG-based measure in schizophrenia [96,97]. Previous studies have highlighted that dysfunctions in the P3b elicitation in schizophrenia might arise from hypo-activation of various brain regions including temporal areas, the cingulate cortex and prefrontal and frontal structures [127,137,138,139,140].

However, despite the vast literature focusing on P3b abnormalities in schizophrenia, findings from previous studies do not allow conclusions on whether these electrophysiological alterations relate to cognitive and/or clinical features of schizophrenia. Our current study revealed that in a large sample of stabilized subjects with chronic schizophrenia, lower P3b amplitude reflected worse performance in the attention-vigilance test scores, reinforcing the hypothesis that P3b is an electrophysiological index of allocation of attentional resources [92,105,141]. The strength of this finding stems from fact that EEG indices were analyzed in a large sample of patients; cognitive functions were evaluated with the MCCB, which is regarded as the gold standard in assessing cognitive impairment in subjects with schizophrenia, and that this outcome was specific of cognitive impairment and not negative symptoms, as documented by the absence of association between P3b and negative symptoms, assessed with a second-generation rating scale [38]. Most of the previous studies have reported a similar linkage between P3b alterations and lower attentive capacities in subjects affected by schizophrenia [102,103,104,105,141]. These data seem to highlight that diminished P3b amplitude values signal an impairment in orientation to task-relevant target information leading to even higher-order deficits in executive processing observed in pathological conditions.

Neuroimaging studies seem to point to a series of alterations in the brain architecture as the possible origin of the attention impairment observed in schizophrenia [142,143,144]. Specifically, disruptions in temporal areas and fronto-limbic circuitry, critical for maintaining an effective interaction between frontal executive and limbic affective processing networks, have been linked to attention deficits [145,146]. Areas involved in these networks, have also been classified as some of the neural generators of P3b, reinforcing the hypothesized link between P3b dysfunctions and alterations in attention circuits [147,148,149].

We also reported a significant association between P3b and aging both in subjects with schizophrenia and healthy controls. This is in accordance with previous results that found a progressive deterioration in P3b elicitation, suggesting that the progressive neural decline observed in aging is likely to deteriorate the neuronal substrates underpinning P3b [101,150,151,152]. The delayed P3b peaks observed in older subjects has been interpreted as an index of slower processing speed, while lower amplitude might reflect the diminished availability of cognitive resources for a given task [99]. Therefore, studies have tried to understand whether changes in P3b only reflect the process of aging or whether these could be used as markers of cognitive decline. One study by Porcaro et al. seems to signal that lower P3b amplitude values were reflecting cognitive impairment, independently from the influence of aging [101]. Our study seems to reinforce this hypothesis, since the correlation found in schizophrenia between P3b and attention remained significant even when controlling for age. Furthermore, an important factor to consider is that in our sample no significant relationships were traced between P3b characteristics and neurocognitive domains in healthy subjects, which individually showed close to normative T-scores, with no sign of cognitive impairment. The presence of the correlation only in the pathological condition, supports the hypothesis that P3b could be used as a marker of severity of neurocognitive impairments, independently from aging [105].

With regard to negative symptoms, we did not find any significant correlations between either the expressive or experiential domain and P3b parameters. In this case, the current literature presents contrasting results. Indeed, some studies reported an association between negative symptoms and P3b amplitude in subjects with chronic schizophrenia [118,119,120] and subjects at-risk for psychosis [121,122,123]. Studies that employed LORETA analysis found that negative symptoms were related to P300 current density localized in the left temporal areas [138,153], posterior cingulate [138] and precuneus [138]. Conversely, no significant correlations were recorded in other studies [88,125,126,127]. The differences reported in the current literature might be due to different factors. Firstly, the experimental design could influence the activation of different neuronal circuits, depending for instance on the level of difficulty associated to the task. Furthermore, which negative symptoms are evaluated and which scales are chosen for assessing them might also play a critical role in the variability of the results. In particular, the majority of studies [118,119,120,123,125,126] used the total score of negative symptoms derived from first generation rating scales, which present several limitations, as they include attentional impairment (SANS, PANSS) or aspects related to disorganization (SANS and PANSS), and provide an inadequate assessment of experiential negative symptoms. Our study aimed to overcome these limitations using the BNSS, a second-generation rating scale, recommended for the assessment of negative symptoms [38].

## 5. Conclusions

In conclusion, in line with previous studies, our results suggested that the elicitation of P3b was noticeably affected in subjects with schizophrenia. These deficits were associated with neurocognitive impairments and not to negative symptoms, suggesting that these two aspects of schizophrenia might not share common pathophysiological mechanisms and could affect higher-order functions, such as goal-directed decision-making, through different mechanisms and neuronal pathways. Further studies, using state-of-the-art instruments, homogenous task design and consistent analysis approaches are recommended in order to confirm the linkage of P3b to severity of neurocognitive impairment in schizophrenia. This would represent a step forward in the search of indices related to those aspects that are recognized as major determinants of poor outcome, such as cognitive impairment, and might therefore foster the development of innovative treatment strategies.

## Figures and Tables

**Table 1:** Demographic characteristics, neurocognitive functions and illness related variables.

	SCZ (*n* = 114)	HCs (*n* = 63)	Statistics	
Gender	81 M–33 W	32 M–31 W	χ^2^ = 7.214; ***p* = 0.007**	
	Mean ± SD	Mean ± SD	*t*/U	*p*
Age	36.86 ± 9.39	34.44 ± 12.48	U = 2982.00	0.062
Educational level (years)	12.35 ± 3.02	13.98 ± 4.04	U = 2759.00	**0.0083**
BNSS Expressive Deficit Domain	11.35 ± 7.27	-	-	-
BNSS Experiential Domain	21.11 ± 9.25	-	-	-
PANSS Positive	8.33 ± 4.74	-	-	-
PANSS Disorganization	8.60 ± 3.49	-	-	-
CDSS Total score	3.24 ± 3.92	-	-	-
SHRS Global Parkinsonism	0.86 ± 1.15	-	-	-
MCCB SoP	32.79 ± 10.42	48.79 ± 9.94	*t* = −9.774	**<0.0001**
MCCB AV	40.20 ± 10.27	51.67 ± 10.22	*t* = −6.420	**<0.0001**
MCCB WM	36.33 ± 11.78	50.60 ± 10.12	*t* = −7.189	**<0.0001**
MCCB VrbLrn	37.02 ± 11.03	52.13 ± 7.30	*t* = −10.489	**<0.0001**
MCCB VisLrn	31.86 ± 13.20	47.76 ± 11.19	U = 1033.50	**<0.0001**
MCCB RPS	38.53 ± 11.33	51.03 ± 8.75	U = 1262.00	**<0.0001**

AV: Attention vigilance; BNSS: Brief Negative Symptom Scale; CDSS: The Calgary Depression Scale for Schizophrenia; HCs: Healthy controls; MCCB: MATRICS Consensus Cognitive Battery; PANSS: Positive and Negative Syndrome Scale; RPS: Reasoning and Problem Solving; SCZ: subjects with schizophrenia; SD: Standard Deviation; SHRS: The St. Hans Rating Scale for extrapyramidal syndrome; SoP: Speed of processing; VisLrn: Visuo-spatial learning and memory; VrbLrn: Verbal Learning and memory; WM: working memory. *p* values in **bold** indicate statistical significance.

**Table 2:** Comparison between SCZ and HCs of P3b amplitude and latency.

	SCZ	HCs		
	Mean ± SD	Mean ± SD	U	*p*
P3b Pz Amplitude	9.60 ± 5.09	14.05 ± 6.10	2023.00	**<0.0001**
P3b Pz Latency	347.36 ± 44.83	327.26 ± 33.25	2683.50	**0.0054**

HCs: Healthy controls; SCZ: subjects with schizophrenia; SD: Standard Deviation. *p* values in **bold** indicate statistical significance.

**Table 3:** Correlations between P3b and clinical/cognitive variables in SCZ.

		SoP	AV	WM	VrbLrn	VisLrn	RPS	Age	ED	Exp
P3b Pz Amplitude	Spearman’s correlation coefficient	0.140	0.259	0.114	0.148	0.044	0.067	−0.0170	−0.060	−0.053
	*p* value	0.144	0.0076 *	0.238	0.124	0.650	0.490	0.070	0.533	0.577
P3b Pz Latency	Spearman’s correlation coefficient	−0.027	−0.107	−0.119	−0.223	−0.195	−0.093	0.320	−0.083	−0.037
	*p* value	0.783	0.278	0.216	0.019	0.043	0.338	0.00052	0.387	0.701

SoP: Speed of processing; AV: Attention vigilance; WM: working memory; VrbLrn: Verbal Learning and memory; VisLrn: Visuo-spatial learning and memory; RPS: Reasoning and Problem Solving; ED: Expressive deficit Domain; Exp: Experiential Domain. Significant *p* value thresholds for correlations: neurocognitive domains (*p* < 0.0083); age (0.05); 2 domains of negative symptoms (*p* < 0.025). * The correlation remained significant when controlling for age and years of education.

**Table 4:** Correlations between P3b, age and cognitive variables in HCs.

		SoP	AV	WM	VrbLrn	VisLrn	RPS	Age
P3b Pz Amplitude	Spearman’s correlation coefficient	−0.028	0.151	0.042	−0.086	−0.017	0.064	−0.470
	*p* value	0.827	0.305	0.757	0.532	0.904	0.633	**0.00010**
P3b Pz Latency	Spearman’s correlation coefficient	0.030	0.021	−0.154	−0.154	−0.159	−0.067	0.309
	*p* value	0.819	0.886	0.249	0.262	0.266	0.620	**0.014**

SoP: Speed of processing; AV: Attention-vigilance; WM: working memory; VrbLrn: Verbal Learning and memory; VisLrn: Visuo-spatial learning and memory; RPS: Reasoning and Problem Solving. Significant *p* value thresholds for correlations: neurocognitive domains (*p* < 0.0083); age (0.05). *p* values in **bold** indicate statistical significance.

## Data Availability

All data supporting the findings of this study are available within the article.

## Supplementary Materials

The following are available online at https://www.mdpi.com/article/10.3390/brainsci11121632/s1, Table S1: Correlation coefficients of P3 amplitude and latency z-scores with neurocognition and negative symptoms in subjects with schizophrenia, Table S2: Correlation coefficients of P3 amplitude and latency z-scores with neurocognition in healthy controls.
