# Supplementary material for: Investigating the Relationships of P3b with Negative Symptoms and Neurocognition in Subjects with Chronic Schizophrenia
Source: Brain Sci. 2021 Dec 10;11(12):1632. doi: 10.3390/brainsci11121632 (PMC8699055; doi:10.3390/brainsci11121632)
Supplement: Supplementary file 1 [file brainsci-11-01632-s001.zip › brainsci-1409234-supplementary.pdf]

Investigating the relationships of P3b with negative symptoms and neurocognition in subjects with chronic schizophrenia

Supplementary materials

Control analyses

- Multiple regression analyses
- Comparison between subjects with schizophrenia (SCZ) and healthy controls (HCs) on age-corrected P3b amplitude and latency z-scores
- Correlations of age-corrected P3b amplitude and latency z-scores with neurocognition and negative symptoms
- Table S1. Correlation coefficients of P3 amplitude and latency z-scores with neurocognition and negative symptoms in subjects with schizophrenia
- Table S2. Correlation coefficients of P3 amplitude and latency z-scores with neurocognition in healthy controls
- Comparison between males and females on P3b amplitude in subjects with schizophrenia (SCZ) and healthy controls (HCs)
- Correlation between amplitude and latency of P3b and depression in subjects with schizophrenia (SCZ)

- Multiple regression analyses

Methods:

In order to evaluate if P3b values, age, education and diagnosis could be employed as predictors of neurocognitive functions and if there was a significant interaction between predictors, we performed six separate multiple linear regression analyses using amplitude and latency of P3b, age, education and group as dependent variables and the T-scores calculated for the six neurocognitive domains as independent variables. If any of the dependent variables resulted to be significantly associated with the independent variable, we tested eventual interaction effects between the predictors. In all the regression analyses, all the values for the predictor variables were mean centered by subtracting the mean of that variable from the original values.

Results:

For speed of processing (SoP), group (β=0.556, $p<0.001$), education (β=0.236, $p<0.001$) and age (β=0.133, p=0.026) resulted to have a significant effect on SoP, accounting for by the 41.5% of the variance of the model. A significant interaction effect was detected only between group and education (β=-0.191, p=0.025).

For attention vigilance (AV), 25.6% of the variance of the scores in this domain could be accounted for by group (β=0.383, $p<0.001$), education (β=0.160, p=0.028) and P3b amplitude values (β=0.163, p=0.029). Therefore, an additional regression analysis was performed to test the presence of a significant interaction between the group and P3b amplitude in the model predicting AV scores. No significant *Group X P3b amplitude* (β=-0.037, p=0.692) or other interaction effects ($p>0.05$) between predictor variables were detected within the model predicting AV.

For working memory (WM), values were only predicted by the group variable (β=0.396, $p<0.001$), and education (β=0.292, $p<0.001$), which accounted for by 28.6% of the variance of the model. A significant interaction effect was detected between group and education (β=-0.284, p=0.002).

For verbal learning (Vrb Lrn), results showed that 30.6% of variance in scores in this domain could be accounted for by group (β=0.487, $p<0.001$) and education (β=0.183, p=0.006). A significant interaction effect was detected between group and education (β=-0.250, p=0.007).

For visual learning (Vis Lrn), results showed that 16.7% of variance in the T-scores could be accounted for by the combination of the group variable (β=0.351, $p<0.001$) and P3b latency (β=-0.168, p=0.022) values. The interaction *Group X P3b latency* was not statistically significant for the model (β=-0.059, p=0.485).

For the reasoning and Problem Solving domain, values were only predicted by the group variable (β=0.422, $p<0.001$) and education (β=0.243, $p<0.001$) which accounted for the 27.9% of the variance. A significant interaction effect was detected between group and education (β=-0.212, p=0.025).

- Comparison between subjects with schizophrenia (SCZ) and healthy controls (HCs) on age-controlled P3b amplitude and latency z-scores

Methods:

Age–corrected amplitudes and latencies were derived for each participant to adjust for the effects of normal aging. Specifically, P3b values were regressed on age in the healthy controls (Hamilton et al., 2019; ref. 121 in the main text). The resulting regression equations were used to derive age predicted values that were subtracted from the observed values and divided by the standard-error of regression for both SCZ and HCs participants to obtain amplitude and latency z-scores. These scores reflect deviations from the values expected for a healthy individual of a given age. This approach allows to remove normal aging effects, retaining any pathologic aging effects. Since P3b z-scores were normally distributed, independent sample t-tests were performed to confirm the difference between SCZ and HCs on amplitude and latency values.

Results

T-tests showed a significant effect of group in P3b z-scores both for amplitude ($t = -4.720$, $p < 0.001$) and latency ($t=2.899$, $p<0.01$).

- Correlations between age-controlled P3b amplitude and latency z-scores and neurocognition and negative symptoms

Methods:

Correlations between P3b z-scores (amplitude and latency) and neurocognitive domain scores and negative symptoms (in HCs only neurocognitive domains) were performed using Pearson's or Spearman's rank correlation tests based on data distribution of neurocognitive domains scores and negative symptoms.

Results:

Correlation coefficients are reported in Table S1 and Table S2. Significant correlations ($p<0.05$) were detected only between P3b amplitude and attention vigilance (r_s=0.226) and P3b latency and verbal learning (r_s=-0.204) in subjects with schizophrenia.

Table S1. Correlation coefficients of P3 amplitude and latency z-scores with neurocognition and negative symptoms in subjects with schizophrenia

	SoP	AV	WM	VrbLrn	VisLrn	RPS	ED	Exp
**P3b amplitude**	0.179	**0.226**	0.135	0.093	0.065	0.072	-0.034	0.016
**P3b latency**	-0.100	-0.146	-0,175	**-0.204**	-0.187	-0.095	- 0.075	-0.075

SoP: Speed of processing; **AV:** Attention -vigilance; **WM:** working memory; **VrbLrn:** Verbal Learning and memory; **VisLrn:** Visuo-spatial learning and memory; **RPS:** Reasoning and Problem Solving; **ED:** Expressive deficit Domain**; Exp:** Experiential Domain.

p values in **bold** indicate statistical significance.

Table S2. Correlation coefficients of P3 amplitude and latency z-scores with neurocognition in healthy controls

	SoP	AV	WM	VrbLrn	VisLrn	RPS
**P3b amplitude**	0.073	0.223	-0.097	-0.011	-0.168	0.022
**P3b latency**	-0.015	-0.084	-0.113	-0.067	-0.109	-0.050

SoP: Speed of processing; **AV:** Attention -Vigilance; **WM:** working memory; **VrbLrn:** Verbal Learning and memory; **VisLrn:** Visuo-spatial learning and memory; **RPS:** Reasoning and Problem Solving.

p values in bold indicate statistical significance.

- Comparison between males and females on P3b amplitude in subjects with schizophrenia (SCZ) and healthy controls (HCs)

Methods:

Differences between males and females on P3b features were analyzed separately for SCZ and HCs since the two sample groups (SCZ and HCs) did not have an equal ratio of males and females. Mann-Whitney U Tests were performed to test differences in P3b amplitude and latency based on gender.

Results:

For both groups SCZ (U= 1123.00; p=0.182) and HCs (U=439.00; p=0.433) there was no significant difference based on gender in P3b amplitude. Also for P3b latency, we did not find a significant effect of gender for both sample groups, SCZ (U=1225.50; p=0.488) and HCs (U=401.00; p=0.191).

- Correlation between amplitude and latency of P3b and depression in subjects with schizophrenia (SCZ)

Methods:

Correlations between P3b amplitude and latency and depression (CDSS Total score) were performed using Spearman's rank correlation in subjects with schizophrenia.

Results:

Both for P3b amplitude (r_s=0.126; p= 0.188) and for latency (r_s=0.023; p= 0.812) no significant correlation was detected with depression (CDSS Total) in our sample of subjects with schizophrenia.
